# Engagement of Mesenchymal Stromal Cells in the Remodeling of the Bone Marrow Microenvironment in Hematological Cancers

**DOI:** 10.3390/biom13121701

**Published:** 2023-11-24

**Authors:** Sebastiano Giallongo, Andrea Duminuco, Ilaria Dulcamare, Tatiana Zuppelli, Enrico La Spina, Grazia Scandura, Annalisa Santisi, Alessandra Romano, Francesco Di Raimondo, Daniele Tibullo, Giuseppe A. Palumbo, Cesarina Giallongo

**Affiliations:** 1 Department of Medical, Surgical Sciences and Advanced Technologies “G.F. Ingrassia”, University of Catania, 95123 Catania, Italy; sebastiano.giall@gmail.com (S.G.); palumbo.ga@gmail.com (G.A.P.); cesarina.giallongo@unict.it (C.G.); 2 Division of Hematology, AOU Policlinico, 95123 Catania, Italy; andrea.duminuco@gmail.com (A.D.); annalisa_santisi@hotmail.it (A.S.); 3 Department of Clinical and Experimental Medicine, University of Catania, 95123 Catania, Italy; dulcamareilaria@gmail.com; 4 Department of Biomedical and Biotechnological Sciences, University of Catania, 95123 Catania, Italy; tatiana.zuppelli@gmail.com (T.Z.); enricolaspina@outlook.it (E.L.S.); 5 Department of General Surgery and Medical-Surgical Specialties, University of Catania, 95123 Catania, Italy; gra.scandura@gmail.com (G.S.); sandrina.romano@gmail.com (A.R.); diraimon@unict.it (F.D.R.)

**Keywords:** MSCs, tumor transformation, hematological cancers, senescence, inflammation

## Abstract

Mesenchymal stromal cells (MSCs) are a subset of heterogeneous, non-hematopoietic fibroblast-like cells which play important roles in tissue repair, inflammation, and immune modulation. MSCs residing in the bone marrow microenvironment (BMME) functionally interact with hematopoietic stem progenitor cells regulating hematopoiesis. However, MSCs have also emerged in recent years as key regulators of the tumor microenvironment. Indeed, they are now considered active players in the pathophysiology of hematologic malignancies rather than passive bystanders in the hematopoietic microenvironment. Once a malignant event occurs, the BMME acquires cellular, molecular, and epigenetic abnormalities affecting tumor growth and progression. In this context, MSC behavior is affected by signals coming from cancer cells. Furthermore, it has been shown that stromal cells themselves play a major role in several hematological malignancies’ pathogenesis. This bidirectional crosstalk creates a functional tumor niche unit wherein tumor cells acquire a selective advantage over their normal counterparts and are protected from drug treatment. It is therefore of critical importance to unveil the underlying mechanisms which activate a protumor phenotype of MSCs for defining the unmasked vulnerabilities of hematological cancer cells which could be pharmacologically exploited to disrupt tumor/MSC coupling. The present review focuses on the current knowledge about MSC dysfunction mechanisms in the BMME of hematological cancers, sustaining tumor growth, immune escape, and cancer progression.

## 1. Introduction

**Figure 1:**
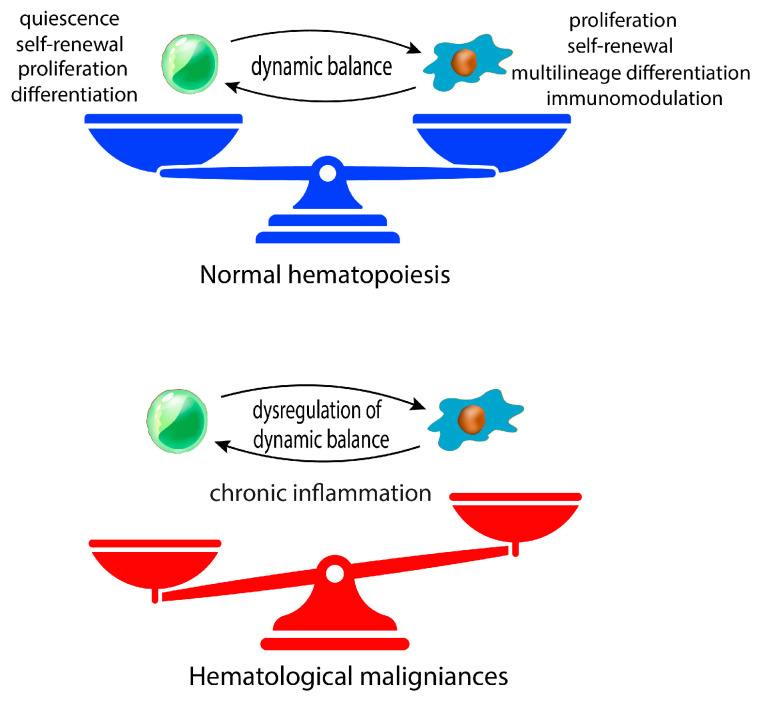
Schematic representation showing the dynamic activity of HSPCs and MSC when the BMME is at homeostasis. The continuous interplay between HSCPs and stromal cells is essential for ensuring normal hematopoiesis. In the hematological cancer scenario, this complex interplay is deeply dysregulated, favoring the trafficking and infiltration of cancer cells into a protective BM niche.

Mesenchymal stromal cells (MSCs) are a critical component of the bone marrow (BMME) microenvironment in which they provide newly formed osteoblasts and tightly regulate the homeostasis of hematopoietic stem and progenitor cells (HSPCs) [1]. In this context, MSCs are the major contributor of many key niche factors and maintain the dynamic balance between HSPC self-renewal, quiescence, proliferation, and differentiation [2,3]. MSCs are located in sites of hematopoiesis, starting from embryonic developmental stages [4]. Importantly, MSCs and their progeny, such as osteoblasts, chondrocytes, and adipocytes, are structural components of both endosteal and perivascular niches [5]. Within these compartments, MSCs interact with both hematopoietic stem cells and more differentiated hematopoietic progenitors, thus regulating their quiescence, proliferation, and differentiation [6] (Figure 1). 

Different MSC subtypes interact with HSPCs in specific regions of the niche [6]: CD271+ MSCs are bone-lining cells sustaining long-term HSPCs in low-oxygen areas, whereas CD146+ and CD271+CD146+ MSCs are located in BM sinusoids with activating and fast-proliferating HSPCs [7]. A plethora of supporting factors regulating HSPC self-renewal and trafficking are provided by MSCs in the BM niche, such as C-X-C motif chemokine 12 (CXCL12) and stem cells factor [8]. Notably, the alteration of HSPC and BM-MSC interactions can alter normal hematopoiesis, causing hematological malignancies [9,10,11]. 

MSC behavior is dynamically regulated both by intrinsic mechanisms and microenvironment factors, highlighting the high plasticity of these cells in adapting to tissue homeostasis and regenerative needs [11]. In this context, the therapeutic use of MSCs in the field of regenerative medicine relies on their ability to migrate to injured tissues and to promote endogenous regeneration, sustaining the growth and differentiation of stem resident cells [12,13]. In details, MSCs’ therapeutic potential for the treatment of immunological diseases results from their ability to suppress or control intensive immune activation by inhibiting immune cell proliferation and inducing immunosuppressive subsets though the secretion of anti-inflammatory factors or direct cell-to-cell contact [13]. However, MSCs also take part in the development of hematological malignancies contributing to BMME malignant transformation and maintenance, finally promoting tumor cell growth, survival, progression, and therapy resistance. Similar to HSPCs, the interactions between cancer cells and BM-MSCs can determinate tumor cell dormancy or proliferation. For example, leukemic stem cells (LSCs) co-localize with CXCL12-secreting MSCs in BM, inducing their quiescence. Furthermore, the accumulation of senescent MSCs in the BM niche might promote the progression from pre- to hematological malignancy [14,15]. The senescence of MSCs is accompanied by several phenotypic changes, including enlarged cell morphology, decreased proliferative capacity, and impaired differentiation ability [16]. Evidence suggests that the presence of increased numbers of senescent MSCs is a characteristic feature of several hematological cancers [17]. When the functional and regenerative capacities of aging MSCs are diminished, they enter a replicative senescence stage which promotes BM inflammation and the dysregulation of hematopoiesis [18] (Figure 1). It is well known that senescent cells release pro-inflammatory factors, generally known as the Senescence-Associated Secretory Phenotype (SASP) which contributes to the tumor immunosuppressive microenvironment [19]. Furthermore, it has been shown that SASP factors such as interleukin 6 (IL-6), C-X-C motif chemokine 8 (CXCL8) and growth differentiation factor 15 (GDF15) can alter HSPC homeostasis in vitro [20]. In detail, IL-6 secreted by aged BM-MSCs induces rapid HSPC expansion, thereby leading to the depletion of the HSPC pool and an increased risk of genomic instability in these cells [14,21]. MSCs are subject to genetic alterations and chromosomal aberrations contributing to age-and disease-associated MSC dysfunctions [21]. Interestingly, it was demonstrated that these alterations differed from the ones observed in the hematopoietic tumor cells of the same patient, corroborating the idea that unstable MSCs might facilitate the expansion of malignant cells [22]. However, no recurring genetic mutations or cytogenetic aberrations have been found in MSCs from the BMMEs of hematological cancers [22,23,24], revealing that epigenetic modifications underlie the activation of their pro-tumor phenotype. In this context, the cellular epigenetic architecture is modeled based on the environmental insults and physiological changes to maintain MSC functions, including their self-renewal, differentiation, and niche-modulation abilities [25]. Notably, dysfunctions of the MSC phenotype also persist after their expansion ex vivo, suggesting a heritable epigenetic dysregulation which persists despite the removal of the disease-associated BMME. In agreement, the methylome of MSCs from hematological cancers was found to be distinct from healthy stromal cells [26,27,28,29].

Data from previous studies revealed that a cancer-associated fibroblast (CAF)-like phenotype is activated in MSCs from patients with hematological cancers [30,31,32]. Indeed, these cells express tumorigenic markers such as alpha smooth muscle actin (αSMA) and fibroblast activation protein (FAP) as consequences of the soluble factors produced by cancer cells [33,34,35]. In agreement, CAFs might derive from MSCs working as a subset of “specialized” stromal cells [36,37]. Paunescu and colleagues previously showed that MSCs and CAF have many similarities, including their phenotype, and the only difference is in the secreted cytokines [38]. In their study, CAFs were demonstrated to derive from a specialization process which converts MSCs inside the tumor structure to better serve cancer cells [38]. A mounting number of studies indicated that growth and survival of leukemic clones is promoted by inflammation-driven changes in BM-MSCs [39]. In particular, naïve MSCs are able to exert a bidirectional effect on tumor cells, favoring or inhibiting their growth, while tumor-“educated” MSCs promote tumor progression in relation to the inflammatory microenvironment [36]. Compared to healthy counterparts, MSCs from the BM tumor milieu show a distinct transcriptional landscape characterized by cellular stress and the upregulation of inflammatory molecules which sustain malignant over healthy clonal hematopoietic cell expansion [23,40]. The pro-leukemic role of MSCs can also be achieved indirectly by shaping the BMME’s immune infiltrate [41,42]. Indeed, the immunomodulatory effect of MSCs on innate and adaptive immunity is a major mechanism through which these cells can affect tumor initiation and progression [43,44,45,46]. This feature depends on the type and intensity of inflammatory signals in the BMME. A high inflammatory state causes MSCs to produce T cell suppression, while a low inflammatory state leads to MSC-induced T cell activation [47]. In hematological malignancies, senescent MSCs release more inflammatory signals, feeding an inflammatory milieu able to scramble the delicate balance of pathways involved in tissue-specific regeneration and remodeling [48]. Although MSC immunomodulatory activity is primed by cytokines in the BMME, it is also dependent on the stimulation of toll-like receptors (TLRs). In detail, MSCs can be polarized into two distinct phenotypes similar to macrophages, resulting in a different immune-modulatory activity and secretome [49]. The TLR4-primed MSCs exhibit a proinflammatory phenotype (MSC1), while the TLR3-primed MSCs activate a more anti-inflammatory profile (MSC2). This concept of MSC polarization could explain the apparently contradictory roles of MSCs in inflammation and immunomodulation [13]. Notably, the regulation of the functional profile of MSCs depends not only on the secretion of soluble factors but also on the communication and contact of MSCs with neighboring BM cells. MSCs can communicate with nearby cells through the secretion of soluble factors, cell-to-cell contact, the release of extracellular vesicles (EVs), and, as evidenced more recently, through tunneling nanotubes [50,51,52,53]. Evidence is arising that altered MSCs help leukemic cell growth and prompt drug resistance by providing nutrients, cytokines, and pro-survival signals and exchanging organelles [32,54,55]. Several recent studies have identified stroma-derived metabolites such as lactate, glutamine, and acetate to feed the tricarboxylic acid cycle (TCA) and lipid biosynthesis into hematopoietic cancer cells [56,57,58]. Of note, metabolism is adjusted during the development of drug resistance [58,59]. The complexity of this scenario is increased by a metabolic heterogeneity and the dynamics of the BMME, which are mainly dependent on differing access to oxygen and glucose and on different cell populations co-existing in the BM milieu [60,61]. As a result, cancer and stromal cells can compete and/or cooperate for nutrients. In recent years, the role of exosomes as mediators between cancer cells and the tumor BMME has gained increasing attention. For instance, leukemia-derived exosomes induced the downregulation of HSPC-supporting factors in MSCs and reduced their capacity to support normal hematopoiesis [62]. Furthermore, while microvesicles (MVs) from healthy MSCs show anticancer action, MM-MSCs release MVs enriched in VLA-4, which facilitates multiple myeloma (MM) cell uptake and enhances the tumor cell phenotype and PC growth [63,64].

In this review, we highlight the role of MSCs in the tumor microenvironment of hematological cancers, aiming to elucidate the mechanisms involved in the activation of their pro-tumor phenotype contributing to tumor growth and progression. 

## 2. Role of MSCs in Hematological Cancers

### 2.1. Role of MSCs in Myelodysplastic Syndromes

**Figure 2:**
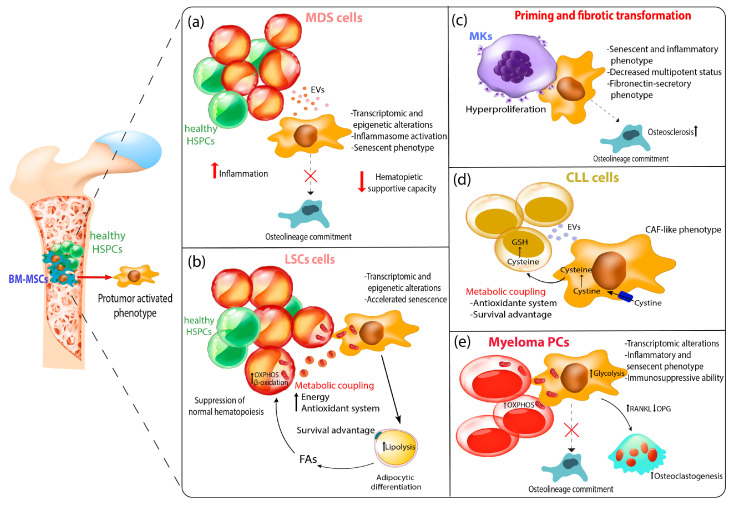
The role of MSCs in different hematological malignancies. The illustration shows how dynamic interactions between malignant cells and MSCs, eventually inducing transcriptomic and epigenetic alterations, affect the stromal secretome and multipotency. (**a**) In MDS, MSCs support the malignant clone at the expense of healthy HSPCs sustaining inflammation and eventually affecting normal hematopoiesis. (**b**) In AML, the interplay between cancer and stromal cells establishes a metabolic coupling leading to immune escape, tumor growth and drug resistance. AML cells promote MSC adipogenic differentiation, enriching the BMME in fatty acids (FA), used by malignant cells by their oxidation. (**c**) In myelofibrosis patients, MSCs drive BM fibrosis differentiating into matrix-producing fibroblasts with increased osteogenic potential, eventually leading to ECM remodeling. (**d**) In CLL context, the metabolic coupling between stromal and cancer cells sustains their antioxidant system maintenance, therefore enforcing the redox balance. (**e**) The metabolic coupling involving malignant and stromal cells has an important role also in MM BMME, where they promote immunosuppression and drug resistance by supporting MM cells oxidative metabolism. Moreover, MSCs contribute to bone disease by prompting osteoclastogenesis over osteogenesis.

Myelodysplastic syndromes (MDSs) are generally referred as a heterogenous group of clonal hematopoietic diseases characterized by ineffective hematopoiesis resulting in peripheral blood cytopenia, potentially shifting to acute myeloid leukemia (AML) [65]. MDS patients display different degrees of cytopenia and dysplasia, therefore constituting the basis for the Word health Organization’s MDS classification criteria [66]. To date, no clinically effective treatment is available for preventing progression to AML. Half of patients show cytogenetic alteration, while nearly 90% of them harbor at least one somatic mutation affecting specific genes involved in the spliceosome, transcription factors, and epigenetics. Despite clonal dominance, these mutations do not provide a determined advantage for malignant cell growth, as suggested by their coexistence alongside normal HSPCs [67]. Therefore, MDS cells receive extrinsic support from the BMME which is important for malignant cell cloning. Notably, support from the BM milieu is essential to maintain MDS cells ex vivo. Concerning MSCs, MDS stromal cells are reprogramed to support uniquely MDS clones at the expense of normal HSPCs [24]. MDS-MSCs are characterized by a slower proliferation rate which is independent of cell cycle distribution and apoptotic events [68]. Cytogenetic aberrations have been difficult to characterize due to the lack of a specific isolation protocol allowing for a comparison between different MSC subpopulations. This goal was achieved when Aanei and colleagues published a robust, immunoselection-based isolation protocol through two specific mesenchymal-associated markers, STRO-1 and CD73 [69]. Therefore, MDS-MSC cytological characterization highlighted genomic gains involving genes taking part in the cell–cell adhesion processes and tumor development. In addition, MSCs isolated from patients harboring 5q-cytogenetic shared common traits, including the overexpression of genomic regions such as 7p22.3, 19p13.3, and 19p13.11 [68]. Although a cytogenetic signature characterizing MDS-MSCs is still missing, it is widely reported that these cells display all the typical markers related to cell senescence [70]. In this context, it has been reported that isolated MDS-MSCs display a profound change in their cytoskeletal architecture, in turn showing an increased size, longer podia, and a disordered distribution of F-actin [71,72]. Moreover, MDS-MSCs also display an increased DNA damage level [71]. Coherently with this outcome, the hyperactivation of p53 signaling cascade was detected in MDS-MSCs, therefore providing a further mechanism leading to MSC senescence [72]. Despite efforts showing the essential role covered by the BMME in MDS, the question asking which is the first cell population to impair basal crosstalk, therefore triggering MDS pathogenesis, still stands. A partial answer was provided by a study describing that in patients showing complete hematological remission, treatment was able to restore MSC functionality comparable to healthy donors [73]. However, this study had the intrinsic assumption that the treatment involves the HSC compartment as the only target, excluding a direct effect on the MSC themselves. In this context, it was recently demonstrated that the antileukemic activity of azacytidine depends, in part, on its direct effect on the hematopoietic supportive capacity of MDS-MSCs, favoring the expansion of healthy over malignant hematopoiesis [74]. These data highlight the crucial role of an epigenetic treatment for dysfunctional MSCs. Other studies corroborate the hypothesis involving crosstalk between the stromal and hematopoietic compartments as a driver of MDS pathogenesis, in turn rearranging the surrounding microenvironment to support the expansion of the malignant clone. Indeed, murine models depleted for Dicer or Sbds gene expression exclusively in the stromal compartment have been shown to develop an MDS-like phenotype characterized by ineffective hematopoiesis, marked dysplasia, and leukemic progression despite having no mutation in their HSPCs [75]. Medyouf and colleagues described a scenario in which MSCs are instructed by malignant HSCs to acquire MDS-MSC-like properties, eventually promoting the progression of the malignant clone over the healthy one [24]. This data introduced the “hematopoietic niche unit”, sustaining MDS progression also via the establishment of an altered secretome profile in which abundant levels of TNF-α, IFN-γ, IL-1α, IL-6, IL-17, and TGF-β have been detected [76]. These factors account for the establishment of an inflammatory BMME, in turn triggering genetic and epigenetic modifications in BM-resident cell populations. Corroborating this, MDS-MSCs show several differentially methylated genes associated to alterations of their phenotype [77]. For instance, the HHIP (Hh-interacting protein) gene is hypermethylated in MDS-MSCs [77]. Its downregulation, accompanied by the activation of the Hedgehog pathway in stromal cells, sustains the survival of MDS cells. Recently, our group showed the relevance of an epigenetic–inflammatory interplay in MDS-MSCs supported by the macroH2A1/TLR4 axis, prompting a replicative senescent phenotype, hypermethylation, and metabolic rewiring, which contribute to ineffective hematopoiesis [78]. In agreement, cellular stress and the upregulation of inflammatory molecules with inhibitory effects on normal hematopoiesis have been described in MDS-MSCs [79]. In particular, the activation of NFkB signaling in MSCs from patients with lower-risk MDS (LR-MDS) attenuates normal hematopoiesis in accordance with cytopenia observed in these patients [79]. Moreover, the overexpression of the alarmins S100A8/9 in the stromal cell compartment has been shown to activate NFkB and a genotoxic stress in HSPCs associated with leukemic evolution in a subset of LR-MDS patients [75]. Supporting the crucial role played by the BM niche in MDS evolution, it has been proposed that the overexpression of CXCL12, in synergy with its receptor CXCR4, keeps myelodysplastic cells anchored inside the BM niche, in turn providing them with protection and support [80]. In this scenario, an in vitro study highlighted the overproduction of IL-6, an interleukin possibly linked to the mechanism promoting MSC senescence and chronic inflammation [81]. The crosstalk between MDS cells and MSCs is also orchestrated by a plethora of factors as part of the two populations’ secretome. By release of alarmins such as S100A9 and S100A8, tumor cells are able to trigger the inflammasome of stromal cells, eventually resulting in a higher secretion of pro-inflammatory cytokines [82]. Also, EVs secreted by MDS cells have been demonstrated to reduce the hematopoietic supportive capacity of MSCs, inhibiting the osteolineage differentiation of MSCs [83]. This perturbation of bone metabolism enables MDS clones to outcompete normal HSPCs (Figure 2a). In turn, MDS-MSCs have been described to release EVs carrying miRNAs, such as miR10a and miR15a, which increase the viability and clonogenicity of MDS cells [84]. Therefore, the multifaced aspects accounting for the significance of MSCs need to be further dissected to provide more efficient strategies for counteracting MDS progression.

### 2.2. Role of MSCs in Acute Leukemia

Acute leukemias are rapidly progressing, malignant clonal disorders characterized by the uncontrolled proliferation of immature and undifferentiated hematopoietic cells and are associated with a poor prognosis and reduced overall survival. They are commonly divided, according to the malignant cells’ lineage, into acute myeloid or lymphoid leukemia (AML or ALL). Blast cells have been known to modify the BMME and disrupt non-malignant hematopoiesis [85,86]. The complex interactions within the tumor BMME significantly influence leukemia survival, disease progression, and therapeutic response, with hematopoietic stem cell transplantation often being the only curative option for patients with refractory disease [87]. Several studies showed that the interaction of leukemic cells with MSCs resulted in a functionally disrupted niche specifically supporting tumor cells over healthy HSPCs and therefore establishing a self-reinforcing unit for the repopulation of leukemic cells [88]. In this context, blast cells might exploit physiological mechanisms regulating hematopoiesis as a strategy for gaining competitive advantages [89]. It has been recently described, using mouse models of leukemia, that both ALL and AML blasts express lymphotoxin α1β2 after colonizing the BM. Therefore, blasts trigger lymphotoxin beta receptor (LTβR) signaling in MSCs, turning off IL7 production and preventing non-malignant lymphopoiesis [89]. Among the changes in the cytokine profile of AML-MSCs, the overproduction of CCL2 inhibits normal progenitors but not leukemic cells, improving cancer survival [90]. Similarly, MSCs from T-ALL patients show a reduced ability to support healthy HSCs, blocking their differentiation in HPCs without direct leukemic MSC-induced damage [91]. This finding is consistent with the paradigm that despite the exhaustion of HPCs in the leukemic milieu, HSCs remain functional upon relocation into a non-leukemic BMME [91]. 

Hematopoietic insufficiency is the hallmark of AML, with cytopenia-related complications such as bleeding and infections representing the major causes of death. In AML-MSCs, the downregulation of FOXM1, a member of the fork-head transcription factor family, impairs the hematopoietic MSCs’ support capacity [92]. Corroborating this, the silencing of this protein in healthy stromal cells affects the growth of CD34+ progenitor cells, mirroring the effects observed when using AML-derived MSCs [92]. Moreover, AML-MSCs displayed alterations in the expression of key hematopoiesis-regulating factors such as JAGGED1 and KITL, corroborating that hematopoietic insufficiency in AML patients is at least in part mediated by the BMME [93]. More recent studies provided evidence that when acute leukemia occurs, blast cells remodel the resident MSCs, establishing a physical connection and mediating a reprogrammed transcriptome [94]. Of note, healthy MSCs changed their gene expression profile after co-culture with AML blasts, displaying the deregulation of genes matched with AML-MSCs [94]. This transcriptomic behavior, characterized by inflammatory factors and cytokine production pathways, correlate to AML, suggesting dynamic changes in MSCs occurring at leukemia onset as consequence of an instructive role of leukemic cells [93,94]. As a result, “reprogrammed” MSCs reset the niche crosstalk processes, selectively suppressing normal hematopoiesis and favoring the clonal dominance of leukemic cells [95,96]. The heterogeneity of MSC subpopulations exhibiting different BMMEs for leukemic cells contributes to the heterogeneous kinetics of leukemia relapse. In this context, Kim and colleagues evaluated whether differences in BM stromal cell partners at diagnosis can identify patients at a high risk of relapse [96]. They found that the BMMEs of relapsed patients showed higher numbers of MSCs, osteoblasts, and primitive nestin+ MSCs than AML patients who achieved complete remission (CR). Early-relapsed patients have a greater primitive MSC content, while late-relapsed ones exhibit more MSCs or osteoblasts than CR patients, corroborating a distinct BMME associated with early or late relapse [96]. This evidence suggests that the leukemia-induced remodeling of the BMME may be responsible for the heterogeneity of the AML clinical course. MSC-lineage differentiated cells, including osteoblasts and adipocytes, are essential components of the BMME contributing to hematopoiesis [97]. Increasing evidence suggests that the differentiation ability of AML-MSCs is altered [98,99]; however, the results are controversial. Indeed, a study reports that AML cells induce an osteoblast-rich niche in the BM which, in turn, enhances AML expansion and favors disease relapse [100]. On the contrary, alterations in MSC osteoblastic plasticity resulted in the selective promotion of leukemic cells in murine models [9,101]. Moreover, other researchers reported that leukemia-educated MSCs are highly prone to adipocyte differentiation [99]. These conflicting results may be due to the heterogeneity of leukemia. For instance, the AML cells of the AML-M4 subtype induce MSCs toward an adipogenic differentiation propensity [102]. Alterations in the osteogenic differentiation capacity of AML-MSCs were also confirmed via specific methylation changes affecting genes regulating cell differentiation and skeletal development [93]. 

As MDS stromal cells, both AML and ALL-MSCs show accelerated cellular senescence, contributing to their impairment in functions associated with HSPC support and stemness properties [19,91]. MSCs exposed to leukemic blasts exhibit characteristics common to MSCs subjected to a physiological aging process, including the overexpression of markers related to DNA damage and cell-cycle arrest [14]. Furthermore, leukemia-induced oxidative stress works as driver of pro-tumoral senescence in stromal cells [103]. Targeting senescent MSCs directly inhibits AML cell growth and improves the survival of mice with leukemia, revealing the importance of a senescent milieu for the pathophysiology of leukemia [103]. Heterochromatin disorganization is a driver of MSC senescence [104]. AML-MSCs downregulate chromatin remodeling complex CHD1 (modulating chromatin condensation), the reduction of which is associated with a decrease in HSPCs’ supportive capacity [105]. Using an integrative approach of multilevel molecular profiling combining genome-wide expression and DNA methylation high-throughput platforms, AML-MSCs were found to exhibit selective transcriptional alterations associated to epigenetic ones, including adhesions molecules, endocytosis, and metabolic pathways [23]. In this context, accumulating evidence shows complex metabolic coupling between leukemic cells and MSCs which allows tumors to respond to variations in nutrient availability to maximize cellular proliferation and acquire survival advantages [106]. In leukemia patients, cancer stem cells or chemoresistant cells rely on mitochondrial oxidative phosphorylation (OXPHOS) [107,108]. MSCs directly provide the increased bioenergetic demand of AML cells, increasing OXPHOS and GSH-related ROS-detoxifying tools which contribute to AML growth and chemoresistance [109]. Of note, MSCs supply mitochondria to leukemia cells [32,110,111,112], thus providing them with additional energy. In T-ALL, leukemic cells transfer their damaged mitochondria to MSCs through cell adhesion mechanisms, reducing intracellular ROS and promoting chemotherapy-induced apoptosis resistance [113]. Recently, it was reported that AML-induced MSCs’ adipogenic differentiation propensity is associated with a switch from glycolysis to OXPHOS [102]. In this context, AML blasts modulate the intracellular metabolism of adipocytes into a lipolytic state, resulting in the release of fatty acids (FAs) into the BMME (Figure 2b) [114]. Ultimately, free FAs are transferred to AML blasts, fueling a FA oxidation signature beneficial to the leukemia counterpart [114]. Indeed, blocking lipolysis or inhibiting CPT1A (carnitine palmitoyltransferase 1a), which is essential for the transfer of FAs to the inner mitochondrial membrane and β-oxidation [115], reduced AML mitochondrial activity and survival [114]. Like AML blasts, ALL cells induce adipocytes to activate lipolysis to support their metabolism [116]. ALL blasts also release EVs which activate a metabolic switch from PXPHOS to aerobic glycolysis in MSCs, leading to increased lactate in the BMME which can be used by tumor cells [117].

### 2.3. Role of MSCs in Myeloproliferative Neoplasms

Myeloproliferative neoplasms (MPNs) are characterized by the clonal proliferation of one or more hematopoietic cell lineages, predominantly in the BMME. MPNs mainly include chronic myeloid leukemia (CML), polycythemia vera (PV), essential thrombocythemia (ET), and primary myelofibrosis (PMF). CML is a BCR-ABL1 oncoprotein-positive MPNs characterized by the Philadelphia (Ph) chromosome’s presence. Ph- MPNs include PV, ET, and PMF, in which clonal proliferation is driven by somatically acquired driver mutations in the JAK2, CALR, and MPL genes [118]. Nevertheless, Ph- MPNs show a different clinical presentation and outcome [119]. PMF is defined by unique clinical features such as BM fibrosis, osteosclerosis, neo-angiogenesis, and extramedullary hematopoiesis which characterize the natural history of PMF patients, significantly affecting quality of life and life expectancy [120]. Similar to MDS-MSCs, stromal cells from CML and higher-fibrosis PMF patients display functional alterations, including low proliferative potential and precocious senescence [121,122]. Changes in MSC behavior are strongly associated with the dysfunction of T cells and the proliferation of Tregs in the CML microenvironment [123]. Moreover, CML-MSCs directly orchestrate immunosuppression by also driving the activation of myeloid cells in MDSCs [41]. The immune suppression of stromal cells can also be enhanced by CML-cell-secreted exosomes [124]. For instance, leukemic-derived exosome miR-130a/b has been demonstrated to promote the immunosuppressive properties of stromal cells through inhibition of connexin-43 [42]. In CML patients, CXCL12-expressing MSCs are crucial for maintaining quiescent leukemic stem cells, and they thus represent a potential target for overcoming drug resistance [125]. Indeed, an analysis of gene expression profiles revealed that abnormal alterations observed in CML-MSCs compared to their normal counterparts persisted in patients in deep molecular response after therapy with tyrosine kinase inhibitors [126], corroborating their role in leukemia relapse and drug resistance. 

Neoplastic clone development in PMF is deeply influenced by alterations within the BBME, highlighted by BM fibrosis, neo-angiogenesis, and osteosclerosis [127,128]. In this context, it may be hypothesized that progressive stromal cell alterations during myelofibrosis evolution affect the disease course [120]. These cells display increased expression and deposition of fibronectin correlating with fibrosis grades [129]. This outcome is further enhanced by megakaryocytes (Mks) aberrantly proliferating and releasing several growth factors mitogenic for MSCs/fibroblasts and endothelial cells, such as TGFβ1 [130]. Corroborating this, in MPN biopsies, MSCs localize with Mks, displaying an activated fibronectin–secretory phenotype [131]. This interaction is crucial for the priming of stromal cells in PMF. Compared to healthy or low-fibrosis-grade MSCs, stromal cells from high-fibrosis-grade PMF patients show a higher capacity to support the differentiation of Mks via fibronectin secretion [129], highlighting their key role in supporting the Mk hyperproliferation observed in PMF BM biopsies [132]. In agreement with this, MSCs isolated from the spleens of MF patients showed higher expression of fibronectin to sustain extramedullary hematopoiesis and megakaryocytopoiesis [133]. In addition to this, other inflammatory molecules generated by malignant clones contribute to the microenvironment abnormalities of the myelofibrosis niche. For instance, lipocalain-2 (LCN2) primes MSCs to differentiate into osteoblasts, prompting matrix protein deposition [134]. Moreover, Mk-derived PDGF activates MSCs and, in particular, the expression of its receptor strongly correlates with the intensity of the MCSs’ reaction and fibrosis grade [135]. We recently demonstrated the involvement of IGFBP6 (insulin-like growth factor-binding protein 6) in the activation of a CAF-like phenotype of stromal cells, controlling the fibrotic process through the activation of the sonic hedgehog/TLR4 axis [136]. Using murine MPN models, Schneider and colleagues demonstrated a critical role for Gli1+ MSCs in the pathogenesis of BM fibrosis [137]. After their activation, dependent on Mk-produced Cxcl4, these cells are metabolically reprogrammed, particularly in fatty acids, and differentiate into matrix-producing myofibroblasts. The authors also demonstrated that the genetic ablation of Gli1+ MSCs abolished BM fibrosis, rescuing BM failure [137]. Of note, an increased number of MSCs can be detected in the blood of PMF patients, suggesting their involvement in abnormal HSPC trafficking/homing leading to extramedullary hematopoiesis [138]. Analyzing the whole transcriptomic profile of MPN-MSCs, Martinaud and colleagues revealed a specific pro-fibrotic and inflammatory signature in PMF-MSCs which is not observed in TE or PV patients and is characterized by increased osteogenic potential and the endogenous production of TGFB1 (Figure 2c) [139]. Leimkuhler et al. found that MSCs transcriptionally downregulated niche support and decreased MSC multipotent progenitor status but upregulated the Mk-derived TGFB1 pathway and extracellular matrix proteins, specifically collagens [140]. MSCs from ET patients were also previously reported to decrease hematopoietic supportive capacity and increase ECM remodeling, suggesting an intrinsic defect of stromal cells already in pre-fibrotic MPNs [131].

### 2.4. Role of MSCs in in Chronic Lymphocytic Leukemia

Chronic lymphocytic leukemia (CLL) is a lymphoproliferative disorder characterized by the relentless accumulation of monoclonal mature B-lymphocytes in the peripheral blood, bone marrow, and lymphoid tissue [141]. A plethora of molecular prognostic factors have been identified in CLL patients and among them, VLA-4, an exclusive member of the α4 integrin subfamily, represents a CLL-negative prognostic marker [142]. VLA-4 plays a prominent role in the homing of high-risk CLL cells within the BMME. Notably, MSC-CXCL12 triggers the activation of VLA-4, therefore highlighting a crucial role played by MSCs in CLL cell homing [143]. Furthermore, MSCs might also promote CLL B-cells resting by increasing their CD38 and CD71 expression, therefore reflecting an activated phenotype that could be related to disease progression [144]. 

In agreement with this, CLL cells highly rely on the abundance of supporting stimuli generated by neighboring cells in the microenvironment, including MSCs. In agreement with this, while CLL cells undergo rapid apoptosis when cultured alone, once cocultured with stromal cells, they are easily propagated. This outcome is probably a consequence of MSC-EVs, which have been recently reported to give to leukemic cells a survival advantage, protecting them from spontaneous and drug-induced apoptosis [145]. In this scenario, CLL-derived exosomes establish a feedback loop by activating a CAF-like phenotype in MSCs, therefore improving the secretion of soluble factors promoting CLL cell survival [146]. Corroborating this scenario, CLL cells isolated from blood samples are non-dividing, although their metabolism is still active [147]. In this context, Jitschin and colleagues reported that CLL cells acquire an increased glucose dependency upon contact with stromal cells [146], in turn promoting glucose uptake in CLL cells by decreasing mitochondrial stress and apoptosis [148]. However, this outcome is still debated. As recently reported, CLL cells co-cultured with MSCs enhance their mitochondrial metabolism, sustaining ATP production along with a nucleotide pool without any change in their proliferation [149]. In agreement with this, it has been reported that CLL cells rely on OXPHOS, and this metabolic process has been associated with poor prognostic outcomes such as IGHV unmutated disease, ZAP70 positivity, increased Rai stage, and higher β2 microglobulin [149]. Therefore, as also described, is possible that leukemic cells modify MSCs’ metabolism to satisfy their energy demand. Recently, it was reported that after contact with CLL cells, MSCs switch their metabolism toward OXPHOS with consequent lower glucose usage, which might be an advantage for CLL survival [148].

As things stand, it might be speculated that MSCs in the CLL context have a crucial role in supporting the malignant clone. In agreement with this, Dig and colleagues demonstrated that the platelet-derived growth factor (PDGF) secreted by CLL cells activates its receptor PDGFR on the MSC membrane [150]. The PDGF/PDGFR interaction enhances MSC proliferation, therefore enhancing the production of VEGF and promoting the neovascularization known to be related to disease progression [151].

Moreover, MSCs uptake cystine by overexpressing the cystine transporter [152]. Upon conversion to cysteine, it is released into microenvironment and internalized by leukemic cells for glutathione synthesis and the maintenance of the redox balance (Figure 2d) [152].

### 2.5. Role of MSCs in in Multiple Myeloma

MM is a hematological disease characterized by the uncontrolled proliferation and expansion of monoclonal plasma cells (PCs) in the BMME that leads to the overproduction of abnormal monoclonal protein and immunoglobulin free light chains. MM evolves from an asymptomatic pre-malignant stage termed monoclonal gammopathy of undetermined clinical significance (MGUS), eventually progressing to an intermediate but more advanced pre-malignant stage defined as smoldering myeloma (SMM) and, finally, to overt myeloma [153]. Although the initiation of the malignant transformation is based on genetic and epigenetic alterations occurring in MM cells, the BMME plays a key role in mediating survival, proliferation, drug resistance, and the progression of the disease [154]. In particular, the interactions of the malignant PCs with other cells in the BM niche, including MSCs, adipocytes, endothelial cells, osteoclasts, osteoblasts, and immune cells, lead to a host of problems including hypercalcemia, anemia, kidney failure, or bone lesions (i.e., the CRAB criteria) [155]. Specifically, mutual modulations of phenotype and functions are observed between PCs and MSCs as a consequence of their bidirectional crosstalking. Bone disease is one of the most prominent clinical symptoms in MM patients, affecting the 80% of MM patients, and seriously impacts the quality of life of patients [156]. As MSCs are osteoblasts progenitors, MM-MSCs actively contribute to the pathogenesis of myeloma bone disease. The adhesion of myeloma PCs to the stroma promotes the tumor cell secretion of several proteins, such as DKK1, which prevents the differentiation of MSCs into osteoblasts [157,158]. Importantly, MSCs not only contribute to bone disease because of their reduced osteogenic potential but also because they ultimately promote the activation of osteoclasts. Interacting with tumor cells, MSCs upregulate RANKL and reduce its soluble receptor OPG, thus prompting osteoclastogenesis through the activation of RANKL-RANK signaling in osteoclasts [159]. 

MM-MSCs exhibit a distinct gene expression profile when compared to MSCs from healthy donors [40,160,161,162]. Particularly, Fernando and colleagues showed that the main downregulated networks in MM-MSCs are related to cell cycle progression, immune activation, and bone metabolism, which might contribute to MM physiopathology [162]. In addition, the expression of specific genes differentiate MGUS-, SMM-, and MM-MSCs, and, interestingly, the gene expression profiles of MSCs from patients with PCs dyscrasias have an independent prognostic impact on clinical outcome [161]. In detail, Schinke et al. identified a prognostic MSC three-gene score, including collagen type IV alpha 1 (COL4A1), natriuretic peptide receptor 3 (NPR3), and integrin beta like 1 (ITGBL1), which is able to predict progression-free survival in MM patients and the progression of MGUS/SMM to MM [161]. Of note, as MSCs from patients who underwent completed treatment show a transcriptome essentially identical to that of patients at diagnosis, persistent printing could maintain a niche prone to relapse [163]. Single-cell sequencing also confirmed that current antitumor therapy fails to counteract MSC inflammation, highlighting their role in disease persistence [164]. MM-MSCs have an early senescent profile characterized by a greater cell size, increased β-galactosidase activity, a Senescence-Associated Secretory Phenotype (SASP), and reduced proliferation due to the accumulation of cells in the S phase [165,166]. This phenotypic change is primed by tumor PCs because healthy MSCs showed a phenotype similar to MM-MSCs after exposure to tumor cells [167]. The senescence of MM-MSCs also impairs their differentiation potential and enhances their tumor-supporting capacity [165]. 

Interestingly, the mechanism behind the establishment of such a phenotype is still unknown. Dicer1, an RNAse III endonuclease essential for miRNA biogenesis, has been demonstrated to be one of the key promoters of cellular senescence in MSCs [11,165]. Specifically, the upregulation of Dicer1 in MM-MSCs reversed cellular senescence and promoted cell differentiation [165]. More recently, Cao et al. provided evidence for a link between MSC senescence and MM progression, investigating genes co-expressed by tumor PCs and MM-MSCs [48]. The authors identified a set of signatures of fourteen genes linked to MSC senescence which are essential in predicting MM progression [168].

Immunosuppression is a common feature of MM associated with disease evolution [169]. Concerning this, our group previously demonstrated that MM-MSCs promote the immunosuppressive abilities of surrounding myeloid cells by promoting the expansion of granulocyte-like myeloid-derived suppressor cells (G-MDSCs) [170] and immunosuppressive neutrophils [46,171], leading to cancer cell immune evasion. As the immunological dysfunction of MSCs was observed already in SMM stromal cells but not in MGUS ones, the activation of an MSC-induced immunosuppressive microenvironment might contribute to the transition from MGUS to MM as an evolutionary advantage acquired during the multistep development of MM. Of note, MSCs from relapsed patients have an increased immunosuppressive ability compared to those from patients in remission [163]. The support of malignant clone proliferation by MM-MSCs is mediated by the stromal activation of the PD1/PDL-1 axis, disrupting T cell immune response [171,172]. Similarly, MM-MSCs are able to induce NK cell exhaustion via the activation of CD155/TIGT signaling [173]. Furthermore, the tumorigenic behavior of MM-MSCs is directly mediated by tumor PCs through the activation of a TLR4-primed inflammatory phenotype [46,171]. Using a single-cell transcriptomic approach, De Jong et al. identified specific inflammatory MSCs in the MM milieu [164]. As successful antimyeloma therapy is unable to revert MSC inflammatory status, not even in patients in which they are undetectable via flow cytometry [174], inflammatory-primed MSCs could be also epigenetically reprogrammed, also maintaining their dysfunction in the absence of tumor cells. In agreement, epigenetic alterations in stromal cells were recently associated with the impairment of bone formation in MM patients [27]. Furthermore, members of the Homeobox family, known as key drivers of osteogenic differentiation, are epigenetically and transcriptionally deregulated in MM-MSCs [27]. Of note, epigenetic alterations in the stromal compartment already occur in the asymptomatic phases of myeloma, and most of these changes are specific to each stage [27]. This phenomenon could be associated to the expansion of MSC subpopulations which promote tumor progression, just as in MM cells [175]. 

The activation of an immunosuppressive and pro-inflammatory phenotype has been associated with a metabolic rewiring of MSCs toward a more glycolytic metabolism which is, in turn, required to sustain the secretion of immunosuppressive factors (Figure 2e) [176]. In agreement, we recently showed that MM-MSCs are more glycolytic than their normal counterparts [55]. Their relative independence from the mitochondrial metabolism impacts MM cell energy, making MM-MSCs inclined to transfer more mitochondria to tumor cells [55]. The uptake of functional mitochondria from MM-MSCs occurs through several mechanisms, including tunneling nanotubes, CD38 [177], and EVs, as well as cell-to-cell contact and the CXCL12/CXCR4 axis [55]. This mitochondrial trafficking supports the oxidative metabolism of tumor PCs, favoring cancer growth and drug resistance [55,178,179,180,181].

## 3. Concluding Remarks and Future Perspectives

MSCs are key components of the BMME, in which they exert multiple functions for supporting the hematopoietic niche, tissue homeostasis, and immune system modulation. The interest in dissecting the role of MSCs in hematopoietic malignancies has vastly grown in recent years. As we discussed above, the BM milieu’s leukemic transformation causes profound modifications in the MSC phenotype, including their morphology and functions with the acquisition of the SASP, which strongly contributes to the development of a proinflammatory microenvironment. Evidence suggests that the SASP-related secretome of MSCs might contribute to the progression from benign states to malignancies [15]. Indeed, the progression of hematological cancers toward a more aggressive phenotype does not solely rely on intrinsic leukemic cell factors but is independently impacted by the biology of the surrounding microenvironment, including MSCs (Figure 2).

Reprogrammed stromal cells provide a nurturing niche that sustains tumor growth, clonal evolution, and drug resistance. Although it has been reported that MSCs from AML patients at the time of disease remission recover healthy activities [94], the inheritance of epigenetic alterations associated with MSC imprinting could lead to an autonomous status of stromal cells from neoplastic clone. In the “absence/decrease” of/in clonal cells after targeted therapies, the persistence of this pathologic inflamed phenotype of MSCs might be a key component partially explaining disease relapse [182]. Moreover, the importance of the BMME is highlighted by the prolonged time to stabilize engraftment after autologous HSPC transplantation. In this case, the prerequisite for transplant success is the rebuilding of the interplay between the BMME and HSPCs. 

For this reason, targeting the BM niche might represent a valuable novel strategy counteracting blood malignancy. Among the emerging targets, the CXCL12/CXCR4 axis disrupts leukemic cell adhesion to MSCs, mobilizing tumor cells into circulation and increasing drug-induced apoptosis [183,184,185]. In our own previous research, the inhibition of this axis also affected tumor/MSC metabolic coupling, inhibiting mitochondria trafficking [55]. In this context, the importance of the metabolic interplay between stromal and leukemic cells for promoting disease establishment and progression is becoming increasingly clear. Mitochondrial transfer supports leukemic cell bioenergetics and antioxidant defenses, sparing them from the high energetic cost of mitochondrial biogenesis. To understand which metabolic vulnerabilities can be targeted in the leukemic BMME might open new avenues for improving cancer therapy. Recently, niche–calcium homeostasis has been found to be involved in the reprogramming of MSCs into a leukemic niche [94]. Compounds blocking the inward movement of calcium modify the transcriptomic and secretome profile of AML-MSCs, restoring healthy functions [94]. Furthermore, the current focus has also been on age-related changes in MSCs which characterize the development of hematological cancers. For this reason, pharmacological approaches to eliminate senescent cells have been investigated [186]. Concerning MSCs, targeting senescent MSCs has been demonstrated as a possible strategy to recover the hematopoietic supportive capacity of stromal cells, improving the metabolic fitness of HSPCs [187]. Therefore, the utility of senolytic agents as a potential intervention in the context of hematological cancer might be a promising new strategy to both inhibit the pro-tumorigenic effects of inflamed MSCs and improve their hematopoietic supportive capacity.

In the framework of the BMME, the complex interplay between leukemic cells and MSCs include dynamic cell–cell interactions and organizations, the release of soluble factors and EVs, and immunoregulatory properties which hide unrecognized leukemogenic events with innovative treatment opportunities. Therefore, extended investigations into the relationships occurring in the leukemic niche may revolutionize treatment strategies to disadvantage cancer cells using niche-directed therapies.
